# The role of fecal microbiota transplantation in diabetes

**DOI:** 10.1007/s00592-025-02508-0

**Published:** 2025-04-19

**Authors:** Gabriele Angelo Vassallo, Tommaso Dionisi, Vittorio De Vita, Giuseppe Augello, Antonio Gasbarrini, Dario Pitocco, Giovanni Addolorato

**Affiliations:** 1 Department of Internal Medicine, Barone Lombardo Hospital, Canicattì, Italy; 2 Internal Medicine and Alcohol Related Disease Unit, Columbus-Gemelli Hospital, Fondazione Policlinico Universitario A. Gemelli IRCCS, Rome, Italy; 3 Department of Medical and Surgical Sciences, Università Cattolica del Sacro Cuore, Largo Francesco Vito 1, 00168 Rome, Italy; 4 Section of Hygiene, Department of Life Sciences and Public Health, Università Cattolica del Sacro Cuore, Rome, Italy; 5 CEMAD Digestive Disease Center, Fondazione Policlinico Universitario A Gemelli IRCCS, Rome, Italy; 6 Diabetes Care Unit, Institute of Endocrinology, Università Cattolica del Sacro Cuore, Fondazione Policlinico Universitario Agostino Gemelli IRCCS, Rome, Italy

**Keywords:** Fecal microbiota transplantation, Intestinal Microbiome, Diabetes, Insulin sensitivity, Metabolic syndrome, Beta-cell

## Abstract

Fecal microbiota transplantation (FMT) has emerged as a potential therapeutic strategy for modulating gut dysbiosis in diabetes mellitus. This review critically evaluates preclinical and clinical evidence on FMT in type 1 (T1D) and type 2 diabetes (T2D). Studies suggest that FMT can restore microbial diversity, improve glycemic control, and modulate immune responses, with varying effects across diabetes subtypes. In T1D, preclinical models demonstrate that FMT influences regulatory T-cell expansion and β-cell preservation, though clinical translation remains limited. In T2D, FMT has shown transient improvements in insulin sensitivity, with sustained effects observed only in patients with specific microbiome signatures. However, heterogeneity in patient responses, donor variability, and methodological limitations complicate its clinical application. This review highlights the interplay between FMT, immune modulation, and microbial metabolism, advocating for phenotype-stratified trials and multi-omics integration to enhance therapeutic precision.

## Introduction

The gut microbiota plays a pivotal role in host metabolism, immune regulation, and disease pathogenesis. Dysbiosis—an alteration in microbial composition and function—has been linked to both T1D and T2D, though the exact mechanisms remain under investigation [1, 2]. 

FMT, the transfer of donor microbiota into a recipient’s gastrointestinal tract, has gained attention as a strategy to restore healthy gut microbiome and influence metabolic pathways [3]. The first report of FMT in clinical practice dates from 1958, when Eiseman successfully treated patients with pseudomembranous colitis, who had a surgical indication, with fecal enemas [4]. While it is an established treatment for Clostridioides difficile infections, its role in metabolic diseases, like diabetes, remains under investigation [5, 6]. 

Gut microbiota is involved in the pathogenesis and progression of both type 1 and type 2 diabetes [1]. 

A landmark study by Wu et al. demonstrated that T2D is characterized by reduced butyrate-producing bacteria (e.g., Roseburia, Faecalibacterium prausnitzii) and an increased abundance of opportunistic pathogens [7]. In T1D, longitudinal cohort studies indicate that microbial disturbances precede autoantibody development, with notable reductions in Bifidobacterium and Akkermansia [8]. 

Diabetes is characterized by lower bacterial diversity, and is associated with a switch toward bacterial strains with pro-inflammatory properties, which may play a role in beta cell loss and insulin sensitivity [7, 9]. Even though the data are conflicting, most findings suggest an increase in Bacteroidetes and a decrease in Firmicutes, with consequent alterations in the ratio of the two phyla, in both types of diabetes. Moreover, a changes in Proteobacteria has been found only in patients with type type 1 diabetes [8]. Finally, both type 1 diabetes and type 2 diabetes patients present a lower level of butyrate-producing microbiota, such as *Roseburia* and *Faecalibacterium prausnitzii* [10] with consequently reduced energy expenditure and mitochondria function usually activated by butyrate [10]. 

FMT exerts critical immunomodulatory effects on diabetes pathogenesis. In T1D, FMT enhances regulatory T-cell (Treg) activity, suppressing autoimmune destruction of β-cells by inhibiting effector T-cells (e.g., Th1/Th17) [11]. This aligns with findings that FMT preserves residual β-cell function in new-onset T1D patients, correlating with reduced islet autoantibodies and IL-17 A levels [11]. In T2D, FMT lowers systemic inflammation by reducing pro-inflammatory cytokines (TNF-α, IL-6) and lipopolysaccharide (LPS) translocation, thereby improving insulin sensitivity [12]. Additionally, FMT restores butyrate-producing bacteria (e.g., *Faecalibacterium*), which promote intestinal barrier integrity and Treg differentiation via histone deacetylase inhibition [13]. These immune-metabolic interactions highlight FMT’s dual role in mitigating autoimmunity (T1D) and chronic inflammation (T2D).

The gut microbiota is also involved in developing diabetes complications. Notably, the development of diabetic retinopathy has been linked to intestinal permeability with consequent uveitis-like inflammation and reduction in butyrate-producing bacteria [14]. In addiction, tauroursodeoxycholic acid (TUDCA), a tertiary bile salts, directly affect retinal ganglion cells [15]. Dysbiosis in diabetes also correlates with vascular endothelial growth factor (VEGF) serum level, which is involved in the progression of diabetic retinopathy [16]. Moreover, a preclinical study highlighted the gut microbiota’s role in chronic pain associated with diabetic neuropathy and in its pathogenesis, showing that both lipopolysaccharide and serum cytokines modulate central pain control and neuronal damage in the spinal cord and dorsal root ganglions [17, 18]. 

All these data suggest the involvement of microbiota in the pathogenesis of both Type 1 and 2 diabetes and a possible role of FMT in treating this disease. Diabetes is heterogeneous, with multiple environmental and genetic contributors. While gut microbiota alterations are evident in diabetic patients, their causal role remains debated [1, 2]. Moreover, there is no single “healthy” microbiome—microbial signatures vary across individuals, influenced by diet, genetics, and disease state [9]. Assessing microbiome activity requires moving beyond taxonomic descriptions to functional assessments due to its complexity [7]. ​ This approach is exemplified by Qin et al.‘s metagenome-wide association study, which revealed that patients with type 2 diabetes exhibit not only a moderate degree of gut microbial dysbiosis but also functional alterations, such as a decrease in butyrate-producing bacteria and an increase in pathways associated with sulphate reduction and oxidative stress resistance [19]. 

In the following paragraphs will be critically discussed the current evidence evaluating the therapeutical role of FMT respectively in type 1 diabetes and type 2 diabetes.

## Type 1 diabetes and fecal microbiota transplantation

Many studies have shown as changes in gut microbiota composition are involved in the pathogenesis and progression of type 1 diabetes [20]. Implicated pathogenetic mechanisms are the reduced level of SCFAs (short-chain fatty acids), alteration of gut permeability with consequent translocation of bacterial products, loss of self-tolerance and enhancement of antigen presentation, activation of the pro-inflammatory pathway, impairment of beta cell function, and insulin resistance [20]. However, the exact mechanism that leads from all these changes to an organ-specific autoimmune disease, such as type 1 diabetes, is still unclear.

### Preclinical evidence

The observation that SCFAs-producing microbiota is reduced in type 1 diabetes [11] has led to the belief that restoring the microbiota may slow down the decline of beta cell function. This finding is also supported by the notion that FMT with stool rich in Akkermansia muciniphila (an SCFA-producing strain) successfully reduced type 1 diabetes prevalence in NOD mice, while FMT without strain selection didn’t [21]. 

## Clinical evidence

Encouraging results of patients with type 1 diabetes who underwent FMT have been reported [22, 23]. A 24-year-old patient with type 1 diabetes and malnutrition underwent FMT for the treatment of nausea, vomiting, and constipation. After FMT, a resolution of gastrointestinal symptoms and an improvement in metabolic parameters have been reported [22]. Two adolescents were treated with FMT one year after the diagnosis of type 1 diabetes. Both patients presented with poor glycemic control. After transplantation, there was a reduction in fasting and postprandial blood glucose levels. HbA1c was also reduced in comparison to the baseline [24]. 

A recent trial investigated the possible role of FMT in this setting, evaluating mixed meal tolerance test (MMTT)-stimulated C-peptide after autologous versus donor FMT in type 1 diabetes patients [24]. Autologous FMT was more effective than donor FMT in preserving the levels of MMTT-stimulated C-peptide at the end of follow-up. However, the study was interrupted before reaching the predicted sample size because of a lack of funding. The results should be taken cautiously and require further investigation [25]. 

## Type 2 diabetes and fecal microbiota transplantation

Preclinical and clinical studies have investigated the link between type 2 diabetes and gut microbiota, evaluating the possible therapeutic role of FMT in patients with type 2 diabetes [26]. 

## Preclinical evidence

A recent preclinical study showed better glycemic control, lower levels of HbA1c, and less suppression of pancreatic beta cell apoptosis in type 2 diabetes mice that had undergone FMT than in those not undergone [27]. On the other hand, a study evaluating the role of synthetic human gut microbiota obtained from diabetic patients and transplanted in mice showed increased body weight and levels of proinflammatory cytokines. Moreover, an increase in LDL, triglycerides and free fatty acids serum levels has been reported in mice. No impact on Oral Glucose Tolerance Test (OGTT) and insuline tolerance test was reported. In this study, gut microbiota was obtained by isolating the seven bacterial strains that were found to be significantly altered in the diabetic patients enrolled [28]. Moreover, in another preclinical study enrolling BTBR^ob/ob^ mice, lower levels of albuminuria were observed in mice that had undergone FMT than the controls, regardless of changes in filtration. These findings support the association between the modulation of gut microbiota and the risk of diabetes-related complications such as diabetic nephropathy [29]. Viruses also play a crucial part in the composition of the gut microbiome and in the pathogenesis of metabolic diseases, leading to a possible role of fecal virome transplantation (FVT) in this field [30]. A study evaluating FVT in diabetes has been performed in DIO mice. Investigators observed that the BMI of mice decreased and OGTT improve after FVT, leading to the hypothesis that weight and glicemic control are mediated through gut microbiota modulation induced by viruses [31]. These preclinical findings provide a basis for further clinical studies on FMT to test its therapeutic potential combined with current treatment for diabetes. However, the influence of antidiabetic medications and diabetes complications (e.g., diabetic gastroparesis) on gut microbiota [14, 17, 18] makes the evaluation of the role of FMT in these patients trickier.

## Clinical evidence

A study enrolled 17 patients with type 2 diabetes for a non-blinded, one-armed intervention trial of FMT. Metabolic parameter changes and gut microbiota composition characterized by 16 S rRNA gene amplicon sequencing from fecal samples were evaluated 12 weeks after FMT. A significant decrease in HbA1c% and blood glucose levels has been observed, while a significant increase in postprandial C-peptide levels has been reported [32]. According to the authors, an abundance of *Rikenellaceae* and *Anaerotruncus* in the feces of patients with type 2 diabetes before the intervention may predict which ones would benefit from receiving FMT [32]. A Chinese study compared the effect of diet (the PPW formulation) in a controlled open-label trial with a combination of FMT and diet in 16 T2D patients. The diet and the combination of diet with FMT led to a significant reduction from baseline in weight, fasting blood glucose, HbA1c, and blood pressure levels. The group that underwent FMT showed more rapid improvement in these parameters, with a subsequent rebound that did not reach the initial values. Interestingly, the group undergoing diet and FMT had lower fasting blood glucose and HbA1c levels at baseline. Bifidobacterium increased during the study in both groups and negatively correlated with blood glucose levels, blood pressure, lipid, and body mass index. Sulfate-reducing bacteria (SRB), Bilophila, and Desulfovibrio decreased significantly after both treatments and showed a positive correlation with blood glucose indices. The study, albeit interesting, is limited by its small sample size, the design, dropouts rate, and the length of follow-up (90 days) [33]. In randomized clinical trials performed by NG et al. [34], 61 obese patients with type 2 diabetes were randomly assigned to three parallel groups: FMT plus lifestyle intervention (LSI), FMT alone, or sham transplantation plus LSI. FMT has been performed every four weeks for up to week 12. The primary outcome was the proportion of subjects acquiring ≥ 20% of microbiota from lean donors at week 24. Proportions of subjects acquiring ≥ 20% of lean-associated microbiota at week 24 were 100%, 88.2%, and 22% in the FMT plus LSI, FMT alone, and sham plus LSI groups, respectively. Repeated FMTs significantly increased the engraftment of lean-associated microbiota. The authors concluded that repeated FMTs combined with LSI enhance the level and duration of microbiota engraftment in obese patients with type 2 diabetes. An increase of butyrate-producing bacteria was found in patients who underwent FMT, whether alone or with the LSI. The group of patients subjected to FMT, and LSI showed an increase in Bifidobacterium and Lactobacillus compared with patients who undergone FMT alone. Moreover, this group of patients presented lower liver stiffness, and total and low-density lipoprotein cholesterol levels at week 24 compared with baseline. No differences between the three groups in terms of glycemic control have been reported in the study. These preliminary results are encouraging, but only three studies, two preclinical and one clinical, showed an improvement after FMT in blood glucose levels [27, 31, 32] and HbA1c% [33]. In contrast, the other studies demonstrated improvement only in weight [33], proinflammatory cytokines, lipid metabolism, and liver stiffness [34]. 

## Conclusion

FMT may enhance diabetes treatment by regulating gut microbiota, improving glucose management, and affecting immunological responses. However, clinical application remains limited due to donor variability, inconsistent patient responses, and transient effects. Engraftment challenges, influenced by recipient diet and microbiome composition, further complicate its efficacy. Moreover, functional assessments like SCFAs and bile acid profiling may better indicate FMT success than taxonomic shifts.

To optimize outcomes, personalized approaches targeting dysbiosis severity and microbiome composition are crucial. Adjunct therapies, such as SCFAs supplementation and bile acid modulators, may enhance durability. Future strategies, including engineered bacterial consortia and synthetic microbiomes, could provide more targeted interventions. Integrating functional metagenomics and immune profiling will be key to maximizing FMT’s therapeutic potential in diabetes.

## Abbreviations

FMT: Fecal microbiota transplantation
TUDCA: Tauroursodeoxycholic acid
VEGF: Vascular endothelial growth factor
SCFAs: Short-chain fatty acids
MMTT: Mixed meal tolerance test
FVT: Fecal virome transplantation
OGTT: Oral glucose tolerance test
SRB: Sulfate-reducing bacteria
LSI: Lifestyle intervention
